# Role of Ketamine in Acute Postoperative Pain Management: A Narrative Review

**DOI:** 10.1155/2015/749837

**Published:** 2015-10-01

**Authors:** Brian M. Radvansky, Khushbu Shah, Anant Parikh, Anthony N. Sifonios, Vanny Le, Jean D. Eloy

**Affiliations:** Department of Anesthesiology and Peri-Operative Medicine, Rutgers-New Jersey Medical School, 185 South Orange Avenue, South Orange, Newark, NJ 07103, USA

## Abstract

*Objectives*. The objective of this narrative review was to examine the usage of ketamine as a postoperative analgesic agent across a wide variety of surgeries. *Design*. A literature search was performed using the phrases “ketamine” and “postoperative pain.” The authors analyzed the studies that involved testing ketamine's effectiveness at controlling postoperative pain. Effectiveness was assessed through various outcomes such as the amount of opiate consumption, visual analog scale (VAS) pain scores, and persistent postoperative pain at long-term follow-up. *Results*. While many different administration protocols were evaluated, delivering ketamine both as a pre- or perioperative bolus and postoperative infusion for up to 48 hours appeared to be the most effective. These effects are dose-dependent. However, a number of studies analyzed showed no benefit in using ketamine versus placebo for controlling postoperative pain. While ketamine is a safe and well-tolerated drug, it does have adverse effects, and there are concerns for possible neurotoxicity and effects on memory. *Conclusions*. In a number of limited situations, ketamine has shown some efficacy in controlling postoperative pain and decreasing opioid consumption. More randomized controlled trials are necessary to determine the surgical procedures and administrations (i.e., intravenous, epidural) that ketamine is best suited for.

## 1. Introduction

Postoperative pain is one of the most undesirable experiences for a patient undergoing surgery. Deliberate action should be taken to prophylactically treat the pain. If postoperative pain does develop, it should be managed early and aggressively, because severe pain not only induces a delay in discharge and poorer patient satisfaction, but also can create a hyperalgesic condition known as persistent postoperative pain (PPP). This strains not only the patient, but also the healthcare system as a whole. Recent studies show that PPP has an incidence as high as 40%. Furthermore, 18.3% of patients report that this pain is moderate to severe [1]. Therefore, it is in the anesthesiologist's best interest to be aware of the severity of this problem and of all the pharmacological agents used to prevent and treat postoperative pain. To date, the mainstay of treatment has been the administration of exogenous opioids such as morphine or fentanyl. However, pain is not always fully relieved by such agents, and often patients develop tolerance to them. The ever-increasing doses of opioids are clearly not without their adverse effects. In addition to that, many patients and even some clinicians wrongly believe that addiction can be inevitable after administration of opioids [2].

Recently, interest has focused on the use of N-methyl-D-aspartate (NMDA) receptor antagonists for the management of postoperative pain. In particular, ketamine has been thrust into the limelight both as a standalone drug and as an adjuvant to other analgesics (e.g., morphine, fentanyl, and tramadol) [3]. Ketamine exerts its main analgesic effect by antagonism of NMDA receptors [4]. In doing so, ketamine modulates central sensory processing of pain. In both animal and human studies, ketamine has shown itself to be a potent antihyperalgesic agent. It can counteract opioid-induced hyperalgesia and prevent the development of opioid tolerance [4].

Ketamine was first described in the literature in 1965 [5] and FDA-approved in 1970 [6]. The drug was originally noticed to have anesthetic effects, much like its close relative, phencyclidine, and only later, its analgesic properties did become coveted [7]. Various studies mentioned throughout this review speak of its use for postoperative analgesia. Ketamine has also been used to treat depression [8, 9], complex regional pain syndrome [10], cancer pain [11, 12], alcohol addiction [13], heroin addiction [14, 15], asthma exacerbations [16], wheezing [17], and pain during propofol injection [18]. Adverse effects can include irritability, nightmares, dissociation, headaches, impaired memory, transient elevations in blood pressure and heart rate, urinary tract symptoms, and hepatotoxicity [19].

This review will describe the pharmacology of ketamine, examine its usage as a postoperative analgesic, and discuss its adverse effects and safety profile.

## 2. Chemical and Structural Characteristics

The word “ketamine” is a portmanteau of the words “ketone” and “amine,” speaking of its chemical structure. It is a hydrochloride salt, available in both powdered and aqueous forms, with the molecular formula C_13_H_16_ClNO and a molecular mass of 237.725 g/mol. It is composed of a chlorophenyl ring structure that is bonded to a cyclohexanone ring. Ketamine has a chiral center at the carbon-2 atom of its cyclohexanone ring and therefore it exists as the optical stereoisomers S(+) and R(−)ketamine. Ketamine was previously only available as a racemic mixture but now comes in its two stereoisomer varieties. S(+)ketamine binds NMDA receptors with affinity that is four times greater than that of R(−)ketamine. While the duration of S(+)ketamine is shorter than that of R(−)ketamine, S(+)ketamine has been shown to have an analgesic potency twice as great as the racemic mixture and four times as great as R(−)ketamine [20].

## 3. Pharmacodynamics

The diverse effects and multifaceted mechanisms of action of ketamine have given it the title “nightmare of the pharmacologist” [7]. As there are many high-quality reviews that focus solely on the pharmacology of ketamine [7, 21–23], we will only touch upon the most salient and novel points. Ketamine's predominant effect is NMDA receptor antagonism. It binds noncompetitively to the phencyclidine binding site of NMDA receptors and modifies receptors via allosteric mechanisms. The NMDA receptor is noteworthy in anesthesia because of its role in central sensitization. This complex process involves a nociceptive signal that is potentiated in the peripheral nervous system, causing hyperexcitability in the spinal cord. It can evoke chronic pain and allodynia at surgical incision sites, as well as at sites surrounding incisions. The blunting of central sensitization has played an important role in the prevention and treatment of both postoperative pain and chronic pain [24]. Ketamine has also been used in IV regional anesthesia, further speaking of its peripheral analgesic effects. However, when used as an effective IV regional agent, the concentrations of ketamine were more than 10-fold greater than when used systemically, decreasing the likelihood that systemic administration provides a strong effect on peripheral receptors [25].

Ketamine causes the central effect of anesthesia when it interacts with NMDA receptors in the brain. Brain concentrations of ketamine were directly related to analgesia using an ischemic pain model. Pain activation in the secondary somatosensory cortex, insula, thalamus, and anterior cingulate cortex was all decreased. Functional magnetic resonance imaging (fMRI) studies demonstrated that both pain and ketamine changed brain connectivity in areas involved in endogenous pain modulation. The studies showed that ketamine was responsible for a decrease in connectivity in the brain regions responsible for pain sensing and affective processing [26, 27].

The analgesic effects of ketamine do not end with NMDA receptors. It has been shown to interact with opioid receptors *μ* preferentially in rats [28]. Studies in guinea pig ileum pointed to possible activation of the *κ*-opioid receptor as the site of analgesia [29]. However, subsequent tests performed with naloxone failed to affect the analgesia attributed to the ketamine, and interaction with any opiate receptor was questioned [30]. Other studies showed the reduction in efficacy of ketamine as an anesthetic induction agent in the presence of naloxone, again implying an interaction with the opiate receptor [31]. A 2014 study by Pacheco et al. which used specific opioid receptor blocking agents suggests that interaction with *μ*- and *δ*-opioid receptors is responsible for the central antinociceptive effects of ketamine [32].

Studies show that prevention of opioid tolerance may be another mechanism of pain prevention by ketamine. It has been reported that *μ*-receptor activation by opioids leads to a sustained increase in glutamate synaptic effectiveness at the level of NMDA receptors. Although the mechanisms that allow ketamine to be an analgesic and opiate-sparing agent after opiate exposure remain poorly understood; an important concept that is worth mentioning is being studied in rat models. Rat studies in brain ischemia have found a role of postsynaptic density (PSD), proteins, specifically PSD-95, in potentiating NMDA function and signaling to nitric oxide synthase resulting in chronic and neuropathic pain. Studies show that ketamine may decrease injury-triggered increases in interactions between the NMDA receptor, postsynaptic density protein 95 (PSD95), and protein kinases, thereby reducing nitric oxide-related neuronal injury [33]. This concept may represent a mechanism underlying reduced pain sensitization and opiate tolerance phenomena. Cancer patients who receive chronic opioids often have developed a high level of tolerance, and, in this situation, ketamine can be a useful adjuvant in analgesia [34, 35].

Many other receptors have been shown to play role in ketamine analgesia; however, their mechanisms remain unclear. Once thought to be an opioid receptor, the sigma-receptor has been implicated as a binding site for ketamine. The role of sigma receptors is uncertain; they may modify other receptors such as NMDA receptors.

Needless to say, the effects of ketamine on the human body, both within the realm of analgesia and without, are incredibly complex. Further study is encouraged to fully elucidate the effects of this multifaceted drug on the body's various receptors.

## 4. Applications in Acute Pain Management

### 4.1. Otolaryngologic Surgery

A 2013 study by Jha et al. [36] (Table 1) evaluated the efficacy of ketamine in postoperative analgesia after surgical site infiltration for pediatric patients undergoing cleft palate surgery. Surgical sites were infiltrated with either 2 mg/kg of bupivacaine or 0.5 mg/kg of ketamine. Although pain scores were similar between groups at the 12 h mark, pain scores were lower with ketamine at the 24 h mark. Only 28% of children in the ketamine group required rescue analgesia, compared to 64% in the bupivacaine group. Administration of ketamine also led to statistically significant decreases in sleep disturbances and dysphagia when compared to the bupivacaine group. This allowed for an earlier resumption of feeding with ketamine infiltration.

Eghbal et al. [37] examined the effects of ketamine on postoperative pain and emergence agitation in children undergoing adenotonsillectomy. This randomized clinical trial (RCT) divided children into a placebo (normal saline) group and a ketamine (0.25 mg/kg IV) group. Emergence agitation scores, acetaminophen requirements, and pain scores at all hours were significantly lower in the ketamine group.

A 2014 meta-analysis by Cho et al. [38] reports that, in tonsillectomy in children, preoperative administration of ketamine, either systemically or locally, resulted in significantly decreased pain at 4 hours and decreased analgesic need over 24 hours.

### 4.2. Spinal Surgery

Nitta et al. [39] tested the efficacy of morphine alone, morphine and ketamine, morphine and clonidine, and morphine/ketamine/clonidine in reducing the need for patient-controlled analgesia (PCA) following cervical and lumbar spinal surgery. When used, ketamine was delivered as a high-dose infusion at 2.0 mg/kg/hour. The study demonstrated that oral premedication with clonidine combined with intraoperative administration of ketamine potentiated the analgesic effect of postoperative IV morphine. There was a significant reduction in the amount of PCA requests and in the amount of cumulative morphine delivered via PCA at 24, 36, 48, and 60 hours. This demonstrates ketamine's adjunctive effect when added to other analgesics, synergistically decreasing overall administration of opioids.

Hadi et al. [40] used low-dose IV ketamine as an adjunct to therapy during and after lumbar microdiscectomy surgery. Patients were divided into a control group, a group receiving 1 *μ*g/kg/min perioperatively and a group receiving both 1 *μ*g/kg/min perioperatively and 1 *μ*g/kg/min postoperatively. The patients in the control group required first analgesic demand dose earlier than the ketamine groups. Total morphine consumption, visual analog scale (VAS) pain scores, and rates of nausea and vomiting were significantly lower in the group receiving both peri- and postoperative ketamine versus the control group.

Kim et al. [41] report similar results in lumbar spinal fusion patients: a ketamine 2 *μ*g/kg/min infusion following a bolus dose of 0.5 mg/kg resulted in patients requiring significantly less fentanyl in the postoperative period with no increase in adverse effects [41].

Pestieau et al. [42] evaluated a low-dose perioperative infusion of ketamine in pediatric patients undergoing scoliosis surgery, a procedure in which opioid analgesic requirements are routinely high. The study was a double-blinded controlled trial with one group receiving ketamine and the other receiving a placebo. Patients were given a bolus of 0.5 mg/kg ketamine prior to incision, an intraoperative infusion of 0.25 mg/kg/h, and a postoperative infusion of 0.1 mg/kg/h. Postoperative opioid usage was similar between the two groups as were the reported pain scores.

### 4.3. Orthopedic Surgery

Cengiz et al. [43] performed a randomized double-blinded study to evaluate the effect of low-dose (6 *μ*g/kg/minute) intraoperative ketamine on postoperative pain after total knee replacement surgery. The control group received a placebo. There was a prolonged time to first analgesic request in the ketamine group and a reduction in cumulative morphine consumption at 1, 3, 6, 12, and 24 hours after surgery. VAS scores were also significantly lower in the group receiving ketamine instead of placebo.

A study performed by Guará Sobrinho et al. [44] assessed ketamine's effectiveness as an analgesic agent when delivered intra-articularly during total knee replacement surgery. Authors did not find a significant difference between groups using 0.25 mg/kg S(+)ketamine, 0.5 mg/kg S(+)ketamine and control groups in terms of postoperative pain scores.

### 4.4. Thoracic Surgery

D'Alonzo et al. [45] tested a single 0.5 mg/kg bolus of ketamine delivered intravenously prior to chest wall incision versus placebo. Levels of IL-6 and C-reactive protein (CRP) were compared before and after surgery between the two groups and reported pain scores at 4 and 24 hours postoperatively. IL-6 levels, CRP levels, and postoperative pain scores at both of these times did not vary significantly between the two groups, suggesting that this dosing schedule of ketamine does not have a measurable effect on postoperative pain in thoracic surgery.

Nesher et al.'s 2008 study [46] examined thoracic surgery patients who received PCA boluses of morphine combined with ketamine versus morphine alone. Fifty-eight patients were divided into two groups, one group receiving 1.5 mg IV bolus morphine upon request and the other receiving 1.0 mg morphine and 5 mg ketamine IV bolus upon request. The group receiving the ketamine/morphine combination required only half the amount of morphine as the morphine-only group. At the 36-hour time-point, 10 of the morphine-only patients still required PCA compared to 5 patients in the morphine/ketamine group. Pain scores and postoperative nausea and vomiting (PONV) were also decreased in the ketamine/morphine group.

### 4.5. Ophthalmological Surgery

Abdolahi et al. [47] utilized low-dose ketamine (0.5 mg/kg) during painful ophthalmologic surgeries (i.e., retinal detachments, strabismus, and keratoplasty). Ketamine was administered during anesthetic induction. Recovery time, postoperative pain scores, anesthetic consumption, analgesic requirements, and perioperative additional analgesic were measured across the two groups. There were no differences in any of these variables between the two groups.

### 4.6. Gynecological Surgery

Thomas et al. [54] studied the usage of a single preoperative dose of ketamine before elective gynecological procedures. This prospective study divided patients into a control group, which received normal saline and a ketamine group, which received 0.15 mg/kg of ketamine just before anesthetic induction. Although the ketamine group required slightly less cumulative morphine for postoperative analgesia, this result was not significant. There was also no significant difference in the reporting of pain on the VAS between the two groups. Although there was no difference in the length of postoperative stays, the group receiving ketamine reported significantly greater satisfaction with their pain management.

Bilgen et al.'s 2012 study [55] utilized three different doses of ketamine (0.25, 0.5, and 1 mg/kg) and a placebo to test the effects on postoperative morphine requirements and pain in women undergoing elective Cesarean sections. The primary outcome assessed was cumulative postoperative morphine consumption at 48 hours. This variable, acute pain score and prolonged postoperative pain measured at 2 weeks, 1 month, 6 months, and 1 year were all similar between all groups. Ketamine did not have a significant effect on any of these outcomes.

Suppa et al. [48] performed a similar study in women undergoing elective Cesarean sections, using a 0.5 mg/kg ketamine bolus 10 minutes after birth in addition to a 2 *μ*g/kg/min IV ketamine infusion for 12 hours thereafter. Usage of ketamine reduced morphine requirements at the 4–8, 8–12, and 12–24-hour time points. The patients in the ketamine group showed reduced pain sensitivity at the T-10 dermatome, suggesting an antihyperalgesic effect. At three years after surgery, patients reported no differences in residual pain or dysesthetic symptoms.

### 4.7. Major Abdominal Surgery

Zakine et al. [49] performed a double-blinded study comparing the effectiveness of ketamine administration in two different settings with one control group which received placebo. The first ketamine group received a 0.5 mg/kg bolus perioperatively and a 2 *μ*g/kg/min infusion thereafter for 48 hours; the second ketamine group received a 0.5 mg/kg bolus perioperatively and a 2 *μ*g/kg/min infusion only for the duration of the surgical procedure. All of the patients were given morphine PCA. The authors found that cumulative morphine consumption was significantly lower in the group receiving the 48-hour infusion when compared to the shorter infusion and control groups. VAS scores were measured postoperatively and were significantly lower in the ketamine groups when compared to the control group. As an added effect, while all patients in the study received hydroxyzine preoperatively, the 48-hour infusion group experienced significantly less nausea than the control group (4% versus 27%, *P* = 0.005). It is worth nothing that there were no side effects of the ketamine, psychotomimetic, or otherwise.

Katz et al. [56] analyzed ketamine as a postoperative analgesic in men undergoing radical prostatectomy. Men were double-blinded into 3 different groups, all of which received fentanyl. In addition, group 1 received a preincisional intravenous bolus (0.2 mg/kg) and infusion (0.0025 mL/kg/min) of ketamine, group 2 received the same dosages 70 minutes after incision, and group 3 received no ketamine. The authors found that pain scores did not differ significantly among the groups and follow-up at two weeks and again at six months they showed no significant differences in pain incidence or intensity.

de Kock et al. [50] studied ketamine given both systemically and epidurally to patients undergoing rectal adenocarcinoma resection. All patients received a preoperative epidural bolus and continuous infusion of bupivacaine/sufentanil/clonidine. A control group received no ketamine, and other groups received ketamine at different dosages, administered either epidurally or intravenously. The group that received a ketamine bolus of 0.5 mg/kg and continuous infusion of 0.25 mg/kg/h had significantly lower PCA requirements, smaller hyperalgesic areas, and less residual pain through six months of follow-up.

### 4.8. Systematic Reviews

A 2011 review by Laskowski et al. [51] found a reduction in total opioid consumption and an increase in time to first analgesic request in groups receiving ketamine. 78% of the ketamine treatment groups required less postoperative opioid analgesia and reported less postoperative pain.

A second review by Elia and Tramèr [52] shows significant decreases in pain intensity at rest when ketamine is compared with a control at 6 h, 12 h, 24 h, and 48 hours postoperatively. All trials except one within the review report a significant decrease in morphine consumption when ketamine is used intraoperatively. Average time to first analgesic request had an average improvement of 16 minutes. Regarding long-term follow-up, significantly fewer patients who had received high-dose IV ketamine (bolus 0.5 mg/kg, infusion 0.25 mg/kg/h during surgery) suffered from residual pain at the 6-month mark.

Subramaniam et al.'s 2004 systematic review [34], including 37 studies and 2385 patients, addressed ketamine's effectiveness in reducing opioid consumption for postoperative analgesia. They divided subjects into 4 subgroups based on the route and method of ketamine administration: ketamine as single dose, continuous infusion, PCA, and epidural ketamine with opioids. Low-dose ketamine was found to be safe, and no increase in side effects was suggested. A single bolus dose of ketamine decreased opioid requirements in 7 of 11 studies. Continuous infusion of ketamine decreased IV and epidural opioid requirements in 6 out of 11 studies. Adding ketamine to PCA morphine was not found to be useful. Epidural ketamine showed beneficial effects in 5 out of 8 studies. Among the various methods of IV ketamine administration, continuous IV infusion of ketamine was the most helpful in major surgeries requiring increased postoperative opiate use such as major abdominal surgical procedures and thoracic procedures.

McCartney et al. [53] performed a systematic review that looked at the effects of various NMDA receptor antagonists, including ketamine, on postoperative pain and analgesic consumption. Twenty-four ketamine-based studies met their inclusion criteria; 14 of 24 (58%) demonstrated a positive effect in lowering postoperative pain and/or decreasing analgesic consumption. Dosages used in the analyzed studies ranged from 0.15 to 1 mg/kg.

## 5. Adverse Effects

Ketamine has been reported to be a safe and well-tolerated drug [57, 58]. Despite the benefits and the increase in popularity of using ketamine as both an anesthetic and an analgesic, there are some troubling adverse effects associated with its use. These effects are usually temporary; however, they can be significantly distressing for patients. Therefore, it is paramount to discuss these effects. Common adverse effects include feelings of inebriation, nausea, psychotomimetic effects, and headaches with long-term use, possibly resulting in impaired cognition, memory, and mood [10].

The most common concerns about ketamine as an analgesic agent are related to its mind-altering effects. Katalinic's team addressed these concerns and their review showed that most of the research conducted using subanesthetic doses of ketamine has shown transient increases in the following psychiatric symptoms: positive and negative symptoms of schizophrenia, dissociative symptoms, and manic symptoms. Fortuitously, these symptoms are only present at times of administration and usually disappear within 60 minutes of administration [19]. Quiet, relaxed surroundings contribute to a reduced incidence of these side effects and when ketamine is administered alone, the prophylactic use of a sedative agent such as 3.75–7.5 mg oral midazolam has generally decreased their incidence and severity [4].

Other psychotomimetic effects of ketamine use include feelings of intoxication, increased confusion, lowered inhibition, and perceptual disturbances. In addition, research shows that chronic use of ketamine may impair several memory systems including episodic and working memory. Nonetheless, these effects have been restricted to time of administration and are transient in nature. Additionally, implications of these effects have not been found to be dependent on dose or route of administration [19].

No severe physical symptoms have been reported with the use of low-dose ketamine; however, studies have reported benign effects of lightheadedness, headache, nausea, diplopia, drowsiness, and dizziness. These effects, unlike the psychotomimetic effects, tend to be dose-dependent. They are also limited to time of administration and a short time thereafter [19].

Ketamine can also induce various CNS side effects (e.g., dizziness, diplopia, dysphoria, dreams, hallucinations, disorientation, strange sensations, lightheadedness, sleep difficulties, and confusion). One systematic review found incidences of these CNS effects were 18%, 10%, 9%, and 0.7% with IV PCA, IV infusion, IV single dose, and epidural groups, respectively. No significant increase in these effects was seen compared to patients who did not receive ketamine [34].

Almost 200 cases of ketamine-induced uropathy have been reported in the literature, mostly in the context of chronic abuse, but five within medical analgesic use [19]. Case series demonstrate a temporal link between ketamine abuse and urological symptoms, urinary tract damage, and renal impairment, with some but not all symptoms improving upon cessation of ketamine. Chu et al. reported lower urinary tract symptoms of ketamine-induced cystitis, dysuria, urgency, frequency, incontinence, macroscopic hematuria, and suprapubic pain. Investigations showed negative urine cultures in 97% and cystoscopy showed epithelial damage in 71% [59].

Hepatotoxicity has been reported at anesthetic doses (≥1 mg/kg) and patients receiving low-dose continuous infusion. Noppers et al. present data on the occurrence of ketamine-induced liver injury during repeated administrations of S(+)ketamine for the treatment of chronic pain. Six patients received 2 continuous intravenous 100-hour S(+)ketamine infusions, 10–20 mg/h, separated by 16 days. Three of these patients developed hepatotoxicity evident through elevated transaminases. Ketamine infusion was promptly terminated and the liver enzymes slowly returned to reference values within 2 months, suggesting an increased risk for development of ketamine-induced liver injury when the infusion is prolonged and/or repeated within a short time frame [60]. Trials using single doses of ketamine <1 mg/kg report no changes in liver function tests [19].

It has been described in the literature that ketamine use can lead to neuronal cell apoptosis. This is triggered by the direct blockade of NMDA receptors. The effect was demonstrated in rats at repeated doses of 20 mg/kg [61]. However, an effect was not seen after a single bolus at this dose [62]. This is especially concerning in the developing brain, where ketamine can interfere with both neuronal proliferation and differentiation [63]. Recent studies have already indicated that ketamine can result in long-lasting cognitive deficits in a primate model. Paule et al. tested this effect with rhesus monkeys who received enough ketamine to maintain anesthesia for a 24 hour surgery during their first week of life. At approximately 10 months of age, control monkeys began to outperform the ketamine-dosed monkeys at a battery of tests. At three-and-one-half years of age, the ketamine monkeys still performed worse than the controls [64].

Chang et al. [65] reported a marked and sudden increase in intracranial pressure (ICP) during induction with ketamine in a patient with traumatic brain injury. It should be noted, however, that this patient's ICP was quite elevated even before induction. The ICP fell rapidly when mechanical ventilation began. Administration of ketamine for sedations has been shown to result in higher opening pressures during lumbar puncture in children, but this effect, like the psychotomimetic effects, can be tempered with midazolam [66].

In a study conducted to evaluate the safety and efficacy of ketamine for the treatment of refractory status epilepticus, it was found that the drug's administration caused possible adverse events in 5 out of 60 study patients. These included a life-threatening severe acidosis, a syndrome similar to propofol-related infusion syndrome, supraventricular tachycardia, and atrial fibrillation [67].

Ketamine is not FDA-approved for epidural use as it carries a risk of neurotoxicity. Furthermore, there is no safety or efficacy data on the neuraxial administration of ketamine. Because of this, the epidural and/or intrathecal administration of ketamine are discouraged outside of the preclinical research setting. Spinal neurotoxicity after continuous intrathecal ketamine infusion of 5 mg/day over 3 weeks has been reported [68]. However, the drug used in this case was not preservative-free, and chemical cytotoxicity has often implicated the preservatives used rather than the active compound. Therefore, only preservative-free ketamine should be used for intrathecal infusion. This effect was also described after a 3-week continuous infusion, a situation that we are unlikely to see in patients that are given ketamine with the short-term goal of postoperative analgesia.

Potential dependence through chronic or repetitive ketamine use is concerning. Studies of recreation ketamine-using populations report cravings for the drug, physiological tolerance, and possible withdrawal symptoms on drug cessation. Physiological tolerance is of particular relevance as it has been suggested for individuals having undergone repeated procedures requiring anesthesia with ketamine [19]. Tolerance development with ketamine used as analgesia is less certain. Perry et al. [69] followed healthy subjects given subanesthetic doses of ketamine for up to 6 months. Results showed no reports of cravings or abuse outside of the study, suggesting that ketamine in low doses is less likely to produce dependence.

## 6. Conclusion

Although it was first used purely as an anesthetic, ketamine is making a certain resurgence in the management of postoperative pain. Many of the aforementioned studies have demonstrated significant effectiveness in controlling postoperative pain, increasing time to first analgesic request, and decreasing overall opioids required, as well as demonstrating fewer of the opioid-related side effects like PONV. These effects have been demonstrated across many realms of surgery, including otolaryngology, abdominal surgery, orthopedic surgery, spinal surgery, and gynecological surgery. While all of the exact mechanisms have not been entirely agreed upon, the primary effect of the drug is related to its NMDA receptor antagonism. An intravenous bolus given prior to incision followed by a continuous infusion appears to be the most effective modality for postoperative pain control. If the infusion is given over a protracted time course (48 h) for more invasive and routinely painful procedures, patients can have a decreased risk of developing persistent postoperative pain in months that follow. This infusion can also be combined with PCA for excellent control of acute postoperative pain. Ketamine may not provide a clear benefit for patients undergoing smaller procedures, as analgesia with low dosages of opioids, NSAIDs, and local anesthetic infiltration can usually provide adequate pain relief. The often feared psychotomimetic effects can be blunted by administering general anesthesia or the less effective benzodiazepine. While animal models show some degree of neurotoxicity in high-dose applications and possible induction of neuronal apoptosis, these effects have not been demonstrated and are not of concern at the dosages and durations employed for postoperative analgesia. The other conclusion that can be drawn from the aforementioned studies is that although ketamine was effective in many of the trials, other trials showed that it provided no benefit when compared to a placebo. While ketamine holds a place in the prevention and treatment of postoperative pain, more large, high-quality controlled studies are necessary in order to determine which procedures it is best suited for and at what dosages and frequencies it should be administered.

## Figures and Tables

**Table 1:** Studies in which ketamine usage has significant benefit.

Author	Year	Surgical setting	Dosing	Timing	Outcomes
Single studies					
Jha et al. [36]	2013	Cleft palette repair	0.5 mg/kg infiltration of surgical site		Lower pain score than bupivacaine (2 mg/kg) at 24 h, less rescue analgesia required
Eghbal et al. [37]	2013	Adenotonsillectomy	0.25 mg/kg IV bolus		Decreased emergence agitation, acetaminophen requirements, and pain scores
Nitta et al. [39]	2013	Cervical and lumbar spinal surgery	2.0 mg/kg/h IV	Bolus given for 5 h periop. (total of 10 mg/kg total)	Reduction in PCA requests and total morphine distributed at 24, 36, 48, and 60 hours
Hadi et al. [40]	2013	Lumbar microdiscectomy	1 *μ*g/kg/min IV	Peri- and postop. for a total of 24 h	Decreased total morphine consumption, pain scores, and PONV
Kim et al. [41]	2013	Lumbar spinal fusion	0.5 mg/kg bolus, 2 *μ*g/kg/min IV infusion	Infusion for 48 h postop.	Less fentanyl requirement postop.
Cengiz et al. [43]	2014	Total knee replacement	6 *μ*g/kg/min IV	Periop. only	Reduction in morphine consumption at 1, 3, 6, 12, and 24 h, lower pain scores
Nesher et al. [46]	2008	Thoracic surgery	1 mg morphine and 5 mg ketamine IV-PCA		Morphine consumption and patients requiring PCA at 36 h reduced by 50%; decreased pain scores and PONV
Suppa et al. [48]	2012	Cesarean section	0.5 mg/kg bolus, 2 *μ*g/kg/min IV infusion	Bolus @ 10 min postop. then infusion for 12 h	Reduced pain sensitivity at T-10 dermatome
Zakine et al. [49]	2008	Major abdominal surgery	0.5 mg/kg bolus, 2 *μ*g/kg/min IV infusion	Periop. bolus, infusion for 48 h	Decreased morphine consumption, pain scores, and PONV
de Kock et al. [50]	2001	Rectal adenocarcinoma resection	0.5 mg/kg bolus, 0.25 mg/kg/h IV infusion	Periop. infusion only	Lower morphine requirements, smaller hyperalgesic areas, and less pain at 6 months of follow-up

Systematic reviews and meta-analyses					
Cho et al. [38]	2014	Tonsillectomy	Various	Preop. dosing	Decreased pain at 4 h, decreased analgesic need at 24 h
Laskowski et al. [51]	2011	Various	Various	Various	100% of ketamine groups required less postop. opioids, 78% reported less postop. pain
Elia and Tramèr [52]	2005	Various	Various	Various	Decrease in morphine consumption, longer time to first analgesic request, less pain at 6 months of follow-up
Subramaniam et al. [34]	2004	Various	Various	Various	Single bolus-less opioid consumption in 64% of trials; continuous infusion-less opioid consumption in 55% of trials; epidural infusion-beneficial in 63% of trials
McCartney et al. [53]	2004	Various	0.15–1.0 mg/kg, various routes	Various	Decreased postop. pain and/or decreased analgesic consumption in 58% of trials'

PONV: postoperative nausea and vomiting.
